# Climatic Control of Upwelling Variability along the Western North-American Coast

**DOI:** 10.1371/journal.pone.0030436

**Published:** 2012-01-19

**Authors:** Diego Macias, Michael R. Landry, Alexander Gershunov, Arthur J. Miller, Peter J. S. Franks

**Affiliations:** 1 Department of Ecology and Coastal Management, Instituto de Ciencias Marinas de Andalucía, Consejo Superior de Investigaciones Científicas, Puerto Real, Cadiz, Spain; 2 Integrative Oceanography Division, Scripps Institution of Oceanography, University of California San Diego, La Jolla, California, United States of America; 3 CASPO Division, Scripps Institution of Oceanography, University of California San Diego, La Jolla, California, United States of America; National Oceanic and Atmospheric Administration/National Marine Fisheries Service/Southwest Fisheries Science Center, United States of America

## Abstract

The high biological production of the California Current System (CCS) results from the seasonal development of equatorward alongshore winds that drive coastal upwelling. While several climatic fluctuation patterns influence the dynamics and biological productivity of the CCS, including the El Niño-Southern Oscillation (ENSO), the Pacific Decadal Oscillation index (PDO) and the North Pacific Gyre Oscillation (NPGO), the mechanisms of interaction between climatic oscillations and the CCS upwelling dynamics have remained obscure. Here, we use Singular Spectral Analysis (SSA) to reveal, for the first time, low-frequency concordance between the time series of climatic indices and upwelling intensity along the coast of western North America. Based on energy distributions in annual, semiannual and low-frequency signals, we can divide the coast into three distinct regions. While the annual upwelling signal dominates the energy spectrum elsewhere, low-frequency variability is maximal in the regions south of 33°N. Non-structured variability associated with storms and turbulent mixing is enhanced at northerly locations. We found that the low-frequency signal is significantly correlated with different climatic indices such as PDO, NPGO and ENSO with the correlation patterns being latitude-dependent. We also analyzed the correlations between this upwelling variability and sea surface temperature (SST) and sea level pressure (SLP) throughout the North Pacific to visualize and interpret the large-scale teleconnection dynamics in the atmosphere that drive the low-frequency coastal winds. These results provide new insights into the underlying mechanisms connecting climatic patterns with upwelling dynamics, which could enhance our prediction and forecast capabilities of the effects of future oceanographic and climatic variability in the CCS.

## Introduction

The oceanic regions at continental margins are among the most productive and biogeochemically active environments on Earth, accounting for nearly half of globally integrated oceanic primary production and the bulk of sedimentary carbon burial in a narrow 300-km wide zone, which covers only a few percent of the global ocean area [1]–[3]. The most productive coastal environments are the Eastern Boundary Systems (EBS), in which primary production rates average 3–5 times those of open-ocean waters on an areal basis, but export production, organic carbon reaching the benthos, and accumulation of organic carbon in the sediments can be many times larger [4].

The California Current System (CCS) is one of the four main EBS of the world (along with Humboldt, Canary, and Benguela Currents Systems) and constitutes the eastern boundary of the North Pacific subtropical gyre. This CCS is highly variable both spatially and temporally [5], with mesoscale features such as fronts, eddies and jets that produce steep gradients in physical and biological properties [6], [7]. Time scales of variability include events lasting days to weeks (storm-passage of wind-induced upwelling) as well as seasonal, interannual, interdecadal and longer period variability [8].

The high biological productivity of the CCS (and other EBSs) is fueled primarily by the supply of nutrients through coastal upwelling [9], [10], the result of seasonally prevailing equatorward winds [11] that push near-surface waters offshore through Ekman transport, causing nutrient-rich waters from mid-depths to reach the surface [12], [13], [14]. These processes not only stimulate the marine productivity of the area which supports economically important fisheries [15], [16], but also strongly influence coastal climate, with important impacts on human health [17] and energy demand [18].

Upwelling is not a temporally continuous or spatially uniform process, but varies episodically with upwelling- and downwelling-favorable winds (as well as substantial interannual variability) and with local bathymetric and topographic features of the coastline [20]. Nonetheless, the seasonally varying wind forcing of upwelling is regarded as the main driver of the strong annual cycle in the production dynamics of the CCS [11], [20]–[23].

It is also known that several climatic phenomena influence the CCS on interannual scales such as the El Niño-Southern Oscillation (ENSO) [24], [25], the strength and position of the Aleutian Low atmospheric pressure system [5] represented by the phase of the Pacific Decadal Oscillation index (PDO) [26], and the relative change of sea-surface height represented by the North Pacific Gyre Oscillation (NPGO) [10], [27]. However, the precise mechanisms of interaction linking basin-scale processes and CCS dynamics are still obscure.

Several attempts have been made to relate climatic indices (large-scale, long-term variability) with upwelling dynamics as both should be interconnected by the periodicity, intensity and persistence of alongshore winds. This is usually accomplished by removing the strong annual signal [28] from the upwelling series by computing the anomalies from the climatologic annual cycle [29]–[31]. However, none of these previous studies have found coherent low-frequency signals in the upwelling anomaly patterns or any significant correlations with the most relevant climatic indices listed above.

In the present study, we use a different approach, Singular Spectral Analysis (SSA) to analyze the temporal variability of the CCS upwelling. This procedure decomposes a time series into its main eigenvalues making it possible to reconstruct the principal signals (periodic or not) described by their associated eigenvectors [32]. The main advantage of this methodology is that no prior assumptions of the characteristics of the signals within the main series are made (as, for example is done when using climatologic annual signals to calculate anomalies) and, thus, no information is lost from the time series.

The main goal of the present work is to reveal the low-frequency, long-term fluctuations within the time series of the upwelling intensity throughout the CCS. These signals will then be analyzed separately to assess their characteristics and relationships to different climate indices in order to better understand the long-term variability of the CCS and its connections to basin-scale or global climatic processes.

## Materials and Methods

### California Upwelling Index (CUI)

Most methods to estimate the strength of upwelling in coastal regions rely on the calculation of the Ekman transport due to along-shore winds [30], [33]. In the present study, we use the California Upwelling Index (CUI) as the primary indicator of upwelling intensity. This index gives the magnitude of offshore (positive values) or onshore (negative values) water transport (in m^3^ s^−1^) at a given location integrated along 100 m of shoreline.

**Figure 1:**
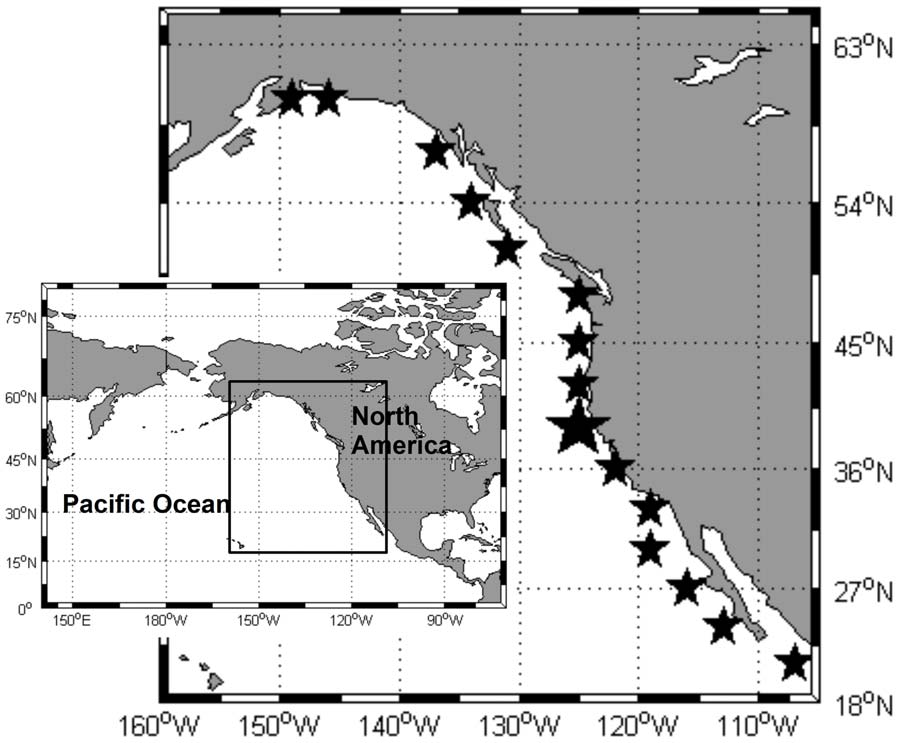
Position of the CUI estimates along the western coast of USA. Inset, map of North Pacific Ocean and box showing the study area.

CUI values are provided by the Pacific Fisheries Environmental Laboratory (PFEL) through the web service available at: http://www.pfeg.noaa.gov/products/las.html. On a monthly basis, PFEL generates indices of the intensity of large-scale, wind-induced coastal upwelling at 15 standard locations along the west coast of North America (Fig. 1) from 21°N to 60°N. The indices are based on estimates of offshore Ekman transport driven by geostrophic wind stress derived from six-hourly synoptic and monthly mean surface atmospheric pressure fields. The pressure fields are provided by the U.S. Navy Fleet Numerical Meteorological and Oceanographic Center (www.fnmoc.navy.mil) Monterey CA [34]. Details regarding the theory and methods used for these transport estimates are given by [35].

### Singular Spectral Analysis (SSA) of the time series

SSA is designed to extract information from short and noisy time series and thus provide insight into the unknown or only partially known dynamics of the underlying system that generated the series [36]. This methodology is analogous to applying an Extended Empirical Orthogonal Function (EEOF) analysis to successive lags of a univariate series, and is equivalent to representing the behavior of the system by a succession of overlapping “views” of the series through a sliding *n*-point window [32]. In so doing, the SSA allows the decomposition of the time series into a sequence of elementary patterns of behavior that are classified as either trends, oscillatory patterns or noise.

The selection of the window size (*n*) is problematic and depends on the objective of the analysis. If *n* is too small, the coarse resolution may cause several neighboring peaks in the spectrum to coalesce. On the contrary, large *n* values (high resolution) will split peaks into several components with neighboring frequencies. So, *n* should be selected depending on the oscillation period one wishes to analyze. There is an upper limit for *n* usually assumed to be the total number of observations divided by 3 [32]; SSA can typically capture oscillations in the range (*n*/5 and *n*). Our chosen value for *n* was 24 months (below the limit established above) and, thus, the extraction range of the SSA includes from 4.5 months to 2 years.

From this decomposition into eigenvalues, it is possible to reconstruct each of the individual signals by adding the corresponding eigenvectors to the sample mean [32]. The Root-MUSIC (Multiple Signal Classification) method [37] was applied to determine the oscillation period of each isolated signal with high resolution.

This SSA method has been satisfactorily used in several oceanographic studies including satellite derived measurements such as SST [38] or chlorophyll-a [39].

### Climatic indices

We compare the temporal variability of coastal upwelling with three basin-scale climate indices with the potential to influence regional behaviors. These are: the Pacific Decadal Oscillation (**PDO**), the North Pacific Gyre Oscillation (**NPGO**) and the Multivariate Enso Index (**MEI**).

The **PDO** index is defined as the leading principal component of North Pacific monthly sea surface temperature (SST) variability (poleward of 20°N), and is connected with the position and strength of Aleutian Low atmospheric pressure system [26]. The PDO term was coined in 1996 by Steven Hare while researching connections between Alaska salmon production cycles and Pacific climate. The PDO “phases” can persist for 20–30 years, and their climatic fingerprints are most evident in the eastern North Pacific/North American sector where they are likely to influence the dynamics and behavior of the CCS. The positive phase represents warm SST anomalies in the eastern North Pacific and cold anomalies extending from Japan to the central North Pacific. The opposite pattern is found during negative phases of the index.

The **NPGO** emerges as the 2^nd^ dominant mode of sea surface height (SSH) variability in the Northeast Pacific [10], [40]. The NPGO has been found to be significantly correlated with previously unexplained fluctuations of salinity, nutrients and chlorophyll-a measured in long-term observations of the California Current and Gulf of Alaska. It was given its name because its fluctuations reflect changes in the intensity of the central and eastern branches of the North Pacific gyre circulation.

The **MEI** monitors the coupled oceanic-atmospheric characteristics of El Niño Southern Oscillations (ENSO) as a weighted average of the main ENSO features contained in the following six variables: sea-level pressure, the east-west and north-south components of the surface wind, SST, surface air temperature, and deep convection as reflected in cloud height. Positive values of the MEI represent the warm ENSO phase (El Niño) and negative the cold phase (La Niña).

### Sea Surface Temperature (SST) and Sea Level Pressure (SLP) data

We obtained large-scale SST and SLP data for the North Pacific from the National Centers for Environmental Prediction–National Center for Atmospheric Research (NCEP–NCAR) reanalysis. These NCEP-Marine data are provided by the NOAA/OAR/ESRL PSD, Boulder, Colorado, USA, from their Web site at http://www.esrl.noaa.gov/psd.

For each grid point within the North Pacific monthly SST and SLP time-series were extracted for the same time-period as the available CUI (1948-2008). Anomalies of both variables (SST and SLP) were calculated by removing the climatologic annual cycles at each grid point.

## Results

### CUI dynamics at 39°N

In this subsection, a complete analysis of the CUI for the 39°N location will be presented. This is intended as an illustration of the analyses performed at the other sites along the coast. The 39°N location (bigger star in Fig. 1) was selected because it is in the middle of the region where coastal upwelling is thought to be the main process governing the local seasonal dynamics [20], [41].

**Figure 2:**
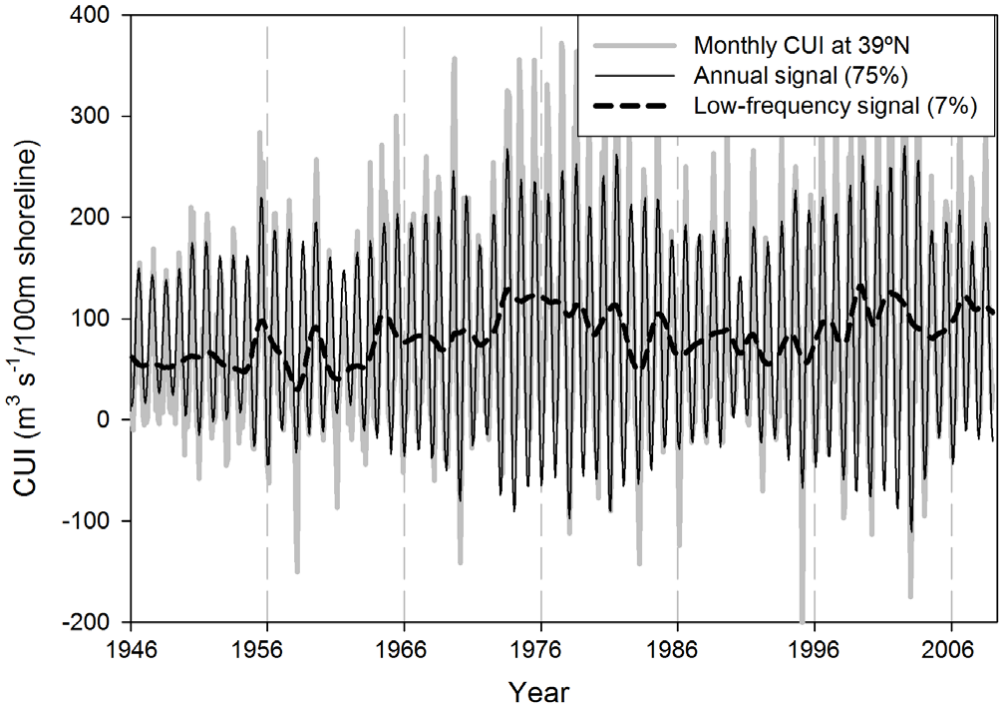
Upwelling index at 39°N. Gray line, monthly CUI; continuous black line, annual signal; broken black line, low-frequency signal.

**Figure 3:**
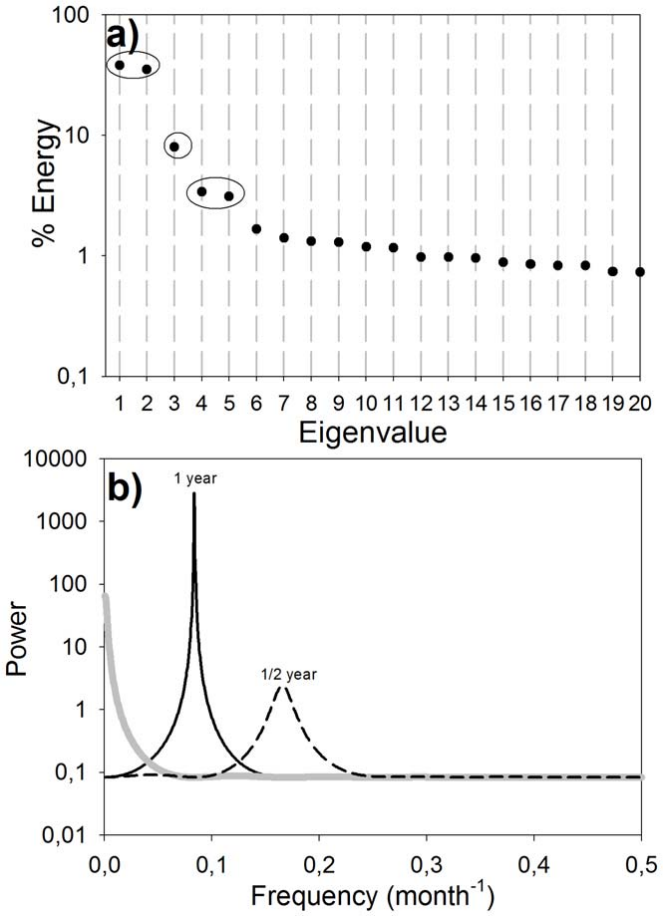
Results of the SSA analysis of the CUI at 39°N. a) Main eigenvalues of the series. b) Three first pure signals; continuous black line, annual signal; broken black line, semi-annual signal; gray line, low-frequency signal.

The CUI time series for 39°N (Fig. 2, gray line) was analyzed for the period 1946 to 2008 (the maximum available data) using the SSA described above. This analysis showed that only the first five eigenvalues of the series were significantly different from noise (Fig. 3a), with the first two presenting very similar energies, the third standing alone and the fourth and fifth again with similar energies. Using the Root-MUSIC method, the combination of the two first eigenvalues gave a signal with annual periodicity (continuous black line in Fig. 3b) that accounted for 75% of total variability of the series (annual signal in Fig. 2). The signal for the third eigenvalue was not periodic (gray line in Fig. 3b) and represented about 7% of total variability (low-frequency signal in Fig. 2). The signal associated with the fourth and fifth eigenvalues showed semiannual periodicity (broken black line in Fig. 3b) and accounted for about 6% of the total variability (Table 1).

**Table 1:** Main signals within the CUI time-series at each location.

Location (latitude)	Annual signal	Low-frequency signal	Semi-annual signal	
21°N	EV	2^nd^ + 3^rd^	1^st^	4^th^ + 5^th^
	% Energy	46.9	33.5	9.7
24°N	EV	2^nd^ + 3^rd^	1^st^	4^th^ + 5^th^
	% Energy	36.5	26.4	20.8
27°N	EV	1^st^ + 2^nd^	3^rd^	4^th^ + 5^th^
	% Energy	51.9	16.2	14.8
30°N	EV	1^st^ + 2^nd^	3^rd^	4^th^ + 5^th^
	% Energy	64.6	10.7	9.1
33°N	EV	1^st^ + 2^nd^	3^rd^	4^th^ + 5^th^
	% Energy	85.9	5.5	3.2
36°N	EV	1^st^ + 2^nd^	3^rd^	4^th^ + 5^th^
	% Energy	82.0	5.2	4.4
39°N	EV	1^st^ + 2^nd^	3^rd^	4^th^ + 5^th^
	% Energy	75.6	6.2	5.7
42°N	EV	1^st^ + 2^nd^	3^rd^	6^th^ + 7^th^
	% Energy	74.5	5.2	4.9
45°N	EV	1^st^ + 2^nd^	3^rd^	4^th^ + 5^th^
	% Energy	69.2	3.9	6.8
48°N	EV	1^st^ + 2^nd^	3^rd^	4^th^ + 5^th^
	% Energy	66.2	4.0	8.1
51°N	EV	1^st^ + 2^nd^	4^th^	3^rd^
	% Energy	46.2	6.0	6.8
54°N	EV	1^st^ + 2^nd^	3^rd^	4^th^ + 5^th^
	% Energy	49.5	5.9	11.2
57°N	EV	1^st^ + 2^nd^	3^rd^	4^th^
	% Energy	65.9	3.7	4.8
60°N	EV	1^st^ + 2^nd^	3^rd^	4^th^
	% Energy	59.6	3.6	6.3

*EV: eigenvectors associated to each signal. % Energy: percentage of variability associated to each signal.*

#### Annual signal

**Figure 4:**
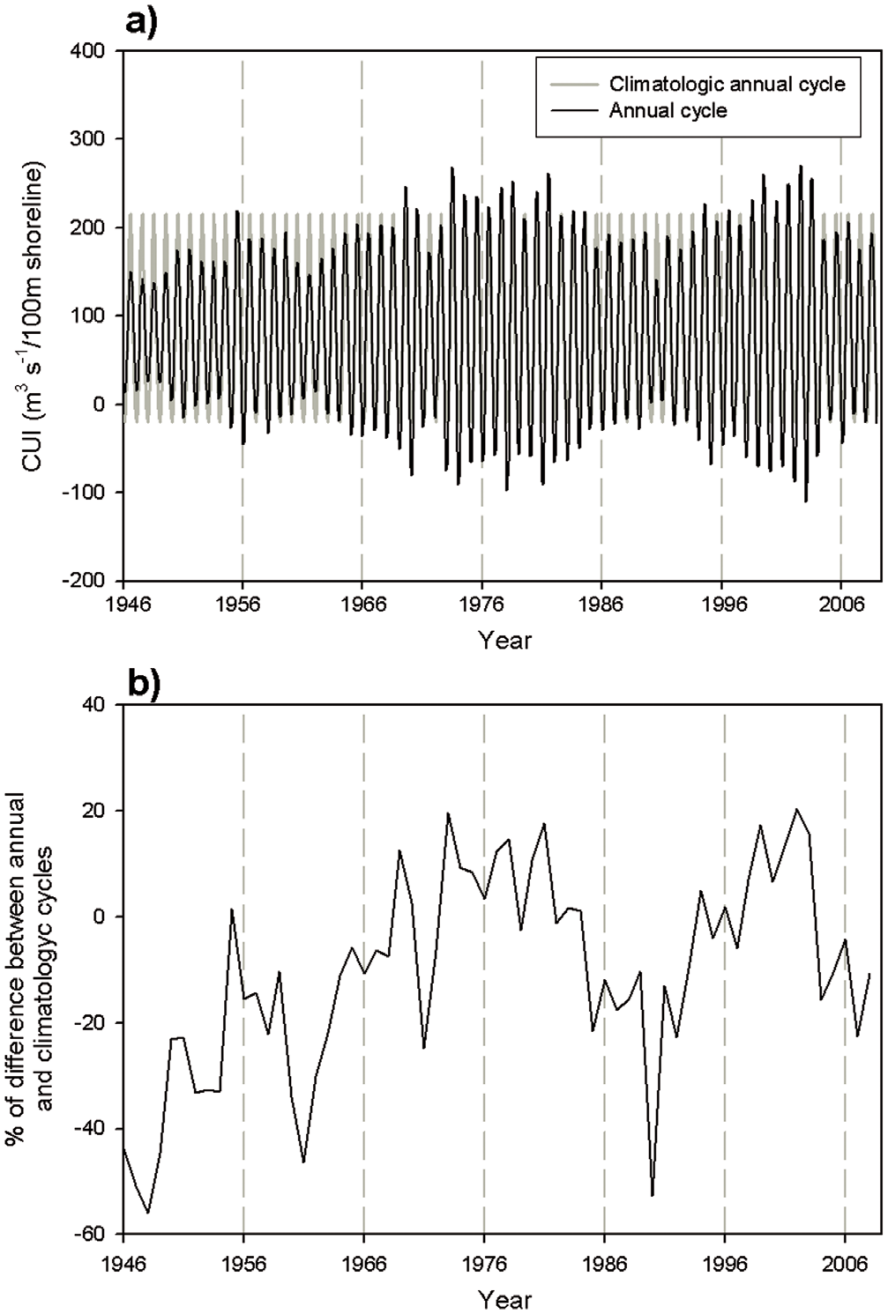
Analysis of the annual signal at 39°N. a) Climatologic (gray) and SSA (black) annual cycles of the CUI at 39°N. b) Percentage of difference between both annual cycles.

The annual signal was the most energetic of all the time series, following the annual cycle of upwelling intensity (Fig. 2) and accounting for more than 75% of the total variability (Table 1). There were appreciable differences between the annual signals from the SSA and the climatological annual signals for the whole time series (calculated as the mean value of the CUI for each month within the series) (Fig. 4a). The differences between the time series accounted for up to 50% of the total value of the CUI in some years, mostly at the beginning of the series (from 1946 to 1966) and during the first half of the 1990s (Fig. 4b).

**Figure 5:**
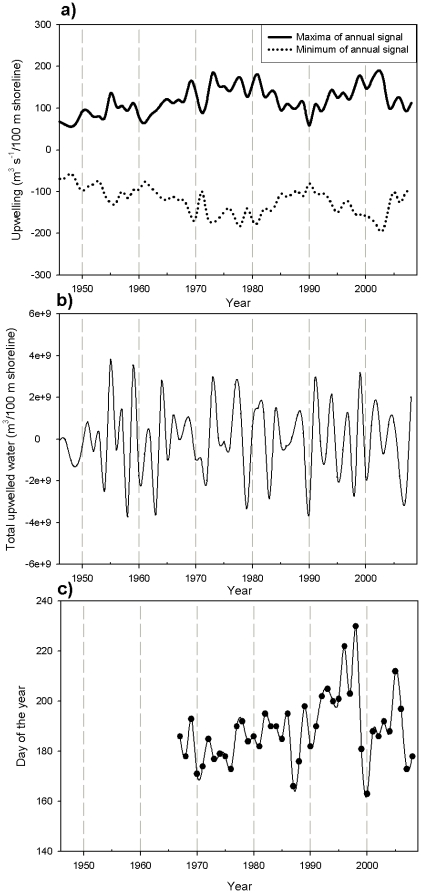
Characteristics of the annual signal of the CUI at 39°N calculated with the SSA. a) Maximum (black line) and minimum (dotted line) values of the annual signal. b) Integrated value of the water upwelled only because of the annual signal. c) Day of the year of the maximum upwelling due to the annual signal.

The SSA also allowed study changes in the amplitudes and phases of the annual signals. For 39°N, the amplitude of the annual signal (Fig. 5a) showed strong interannual variations with a progressive increase (decrease) of the maximum (minimum) values from the beginning of the series to the late 1970s, a subsequent decline (increase) until 1990, and a later increase (decrease) over the decade.

Finally, from the biological point of view, an important parameter of the series is the day of the year when maximum upwelling intensity is achieved within the annual cycle (Fig. 5c). It is clear that the day of maximum upwelling intensity is strongly variable, with interannual differences exceeding 70 days (for example between 1998 and 2000).

#### Low-frequency signal

**Figure 6:**
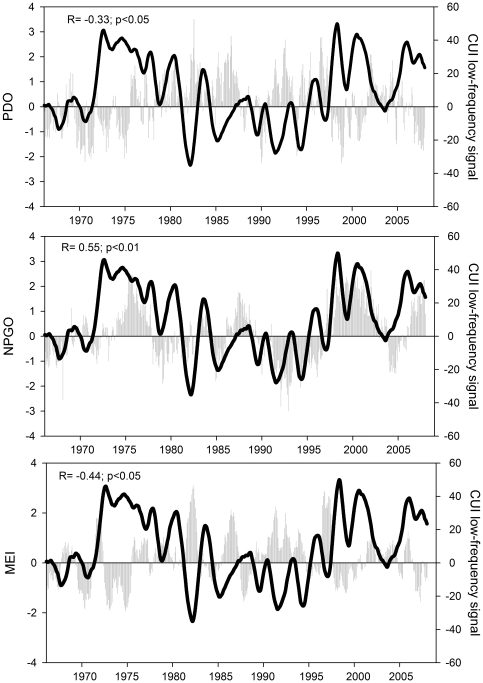
Low-frequency signal of the CUI at 39°N and different climatic indices. PDO (upper panel), NPGO (central panel) and MEI (lower panel). Climatic indices are indicated by gray bars and the low frequency CUI signal as a black line.

The low-frequency signal of the CUI could be compared with the different climatic indices available for this area including the PDO, NPGO and MEI (Fig. 6). The time series of upwelling intensity was negatively correlated with the PDO (Fig. 6a, R = −0.33, p<0.05), positively correlated with the NPGO (R = 0.55, p<0.01) and negatively correlated with the MEI (R = −0.344, p<0.05). For computing all reported significances (in this section and below), the autocorrelation of the time series must be taken into account. For each low-frequency signal, the decorrelation time (*n_d_*), expressed as the number of lags (months) needed for the autocorrelation to drop to zero, has been calculated. Thus, the effective number of degrees of freedom (*n_eff_*) for each comparison is recalculated as:

n_eff_ = N/n_d_

where *N* is the total number of data-pairs to be considered. For each comparison the highest *n_d_* (from the climatic index or from the low-frequency CUI signal) was taken.

### CUI dynamics along the coast

**Figure 7:**
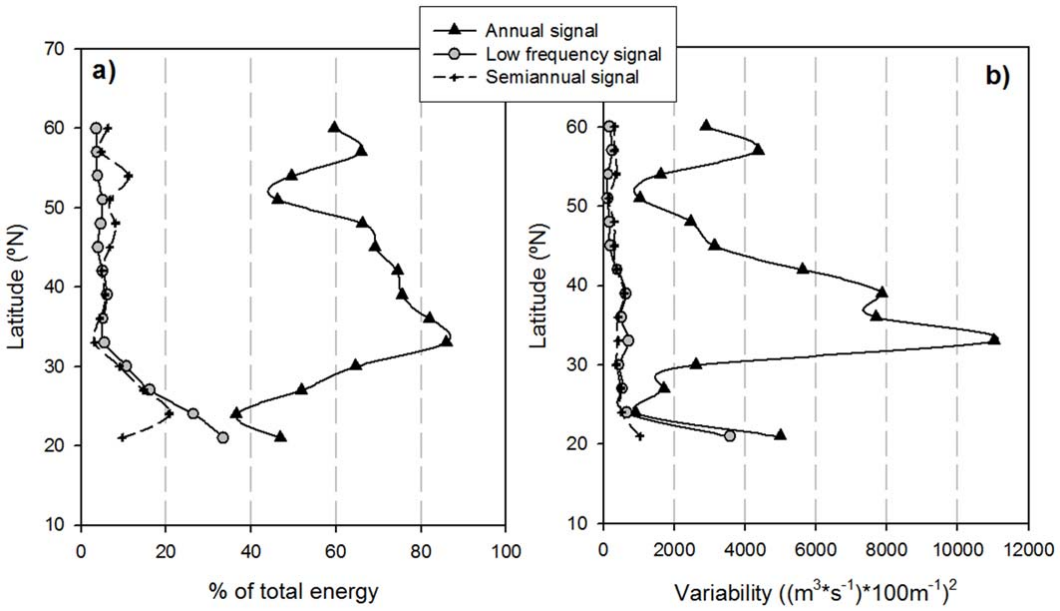
Percentage of energy associated to each signal detected in the SSA analysis. Latitudinal distribution of the energy of the annual (black triangles), semiannual (crosses) and low-frequency (gray circles) signals along the Western North American coast.

The same analytical protocol used above was applied to the CUI time series calculated for the 15 locations along the west coast of North America (Fig. 1, Table 1). The variability associated with each signal (annual, semiannual and low-frequency) at each location showed that the annual signal represented only about 40% of the total variability in the southern part of the region (Fig. 7). The importance of this signal increased up to 87% of total variability around 33–36°N, and remained above 70% until 48°N. From this point northward, its relevance decreased to an average 50% for the rest of the analyzed locations.

The semiannual signal only showed high energy at two locations (24°N and 27°N, mean >15% of total variance), while elsewhere it represented about 5–6% of the total variance. The low-frequency signal is notable in showing the opposite behavior to the annual cycle, being relatively more important at the southern locations (>20% of total variability). Variability explained by the low-frequency signal declined progressively to 6% of total variability at 33°N and remained at this level through the northern stations.

The absolute amount of variability associated with each signal (Fig. 7b) shows a very similar latitudinal pattern, with the annual signal being more relevant at mid latitudes and the low frequency in the southern region. However, differences in the total variability of the CUI time-series between locations enhance some of the latitudinal gradients of the various signals, particularly the annual one.

**Figure 8:**
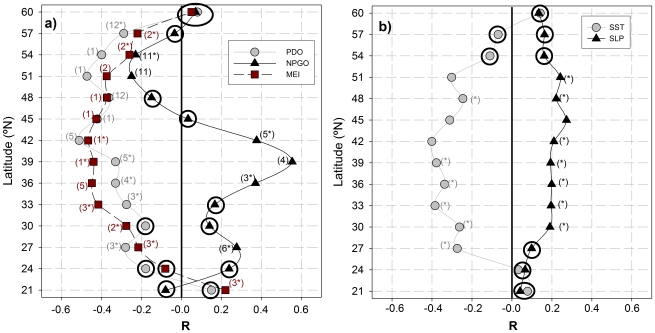
Correlation coefficients of the different low-frequency signals of the CUI with the climatic indices (a) and SST and SLP anomalies (b) along the western US coast. Panel a) Gray circles, PDO; black triangles, NPGO; red squares, MEI. Panel b) Gray circles, SST anomalies; black triangles, SLP anomalies. Insignificant correlations are shown with circled symbols; correlations significant at 0.05>p>0.01 are presented with an asterisk, and the rest are significant at p<0.01.The number in brackets is the month of the year in which the maximum correlation between both series is achieved.

As a final analysis, we computed the relationship between climatic indices (PDO, NPGO and MEI) and the low-frequency CUI signal at each location (Fig. 8a), as done above for the 39°N estimations (see Fig. 6). We also analyzed for seasonality in the strength of the correlations by computing and comparing the correlations for individual months. The month for which maximum correlation was found is presented in brackets beside each R-value (computed using the entire time-series) in Fig. 8.

Correlations with all climatic signals showed strong latitudinal variations, but there were appreciable differences among the different indices. The PDO was negatively correlated with the low-frequency signal at all but the southernmost location. Correlations were statistically significant at the 99% level (p<0.01) between 42° and 54°N, to the 95% level (p<0.05) between 33° and 39°N, and not significant at the southernmost and northernmost locations. The magnitudes of the correlation coefficients were always above 0.3 (maximum>0.5). The strongest correlations were found in early spring (February to March) from 21°N to 42°N. At other locations, the maximum occurred typically during late fall or winter months (November through January).

The correlations with the NPGO showed a complementary pattern south of 42°N, with a negative correlation at the southernmost location and positive for the rest. Correlations were significant (to the 95% or 99% level) at 27°N and between 36° and 42°N with R_mean_ = 0.32, being strongest during spring and summer (April to August) and at mid latitudes (R_max_ = 0.56 at 39°N). From 42°N north, the absolute values of the correlation coefficients dropped and became negative at all stations (p<0.05). The maximum correlation occurred during late fall (November) at these northern locations.

The pattern of correlation with the MEI was similar in shape to that of the PDO, i.e., positive values at the southernmost station and negative at the rest. The correlations with this index were significant (95 or 99%) at all but two locations (24°N and 60°N), with absolute values above 0.28 in all cases. As with the PDO, the strongest correlations were found at mid-latitude stations (between 33°N and 45°N), where absolute R values were above 0.40. There were also appreciable differences in the month of the year of maximum correlation: the southern stations (21°N–39°N) were more correlated during the spring season (February-May) while the northern stations were strongly correlated during the late fall and winter (November to January).

Finally, the correlation coefficients between the low-frequency CUI signals and the anomalies of SST and SLP were computed for each location (Fig. 8b). There was a negative and significant correlation with SST at the majority of locations along the western US coast. There was also a positive (though weak) correlation between SLP anomalies and low-frequency CUI for the entire coast.

## Discussion

Here we have used Singular Spectrum Analysis to examine the long-term temporal variability in coastal upwelling indices along the West Coast of North America. SSA is able to discriminate between the main signals contained in the original series in a distinctive way that preserves nonperiodic signals and identifies slowly varying changes in periodicities.

Upwelling is known to be a strongly seasonal process in the CCS driven by the development of equatorward winds during early spring [21], [42]. Thus, the predominance of the annual cycle as the most energetic signal of the series (accounting for up to 88% of total variability, Fig. 7) is not surprising.

However, the annual signal at 39°N was markedly different from the climatological annual cycle, with the difference accounting for as much as 50% of the total signal (Fig. 4b) during some periods of the time series. The strong interannual differences in amplitude of the annual signal (Fig. 5a) creates a long-term variability in both the maximum and minimum annual values, as observed for SST by [43] using state-space analysis. However, as both extreme limits showed opposing behaviors, the total amount of upwelled water per year (due to this annual signal alone) did not shown any apparent tendency throughout the time-series (Fig. 5b).

The dramatic change that we observed in the phase of the annual signal has been also reported for the SST annual cycle in this same region [20]. In our data (Fig. 5c), the day of the year of the maximum upwelling intensity of the annual cycle could vary as much as 70 days from year to year, agreeing with the reported variability of the spring transition time [44]. This variation in the onset of upwelling determines whether nutrients reach the photic layer during environmental conditions (i.e., temperature and light) that are still optimal for phytoplankton production but not for secondary producers (i.e., long after zooplankton spawning). The biological implications of episodic perturbations of upwelling timing has been clearly described by [45] for the northern California coast in 2005, when upwelling was substantially delayed (Fig. 5c) and ultimately weaker than average (Fig. 5b). This combined effect resulted in lower phytoplankton production and a strong negative zooplankton biomass anomaly in the California coastal system due to the mis-match of production-consumption cycles.

There was no statistically significant relationship between the modulation of the annual signal and any of the climatic indices considered, either at a monthly time scale or using integrated annual values. This suggests that this fraction of the variability of the CUI time series is controlled by factors not included in the analyzed indices.

The goal of the SSA analysis was to remove the fraction of low-frequency variability that is driven by the low-frequency modulation of the seasonal cycle and retains only the “pure” low-frequency dynamics to allow exploration of how large-scale low-frequency Pacific climate controls upwelling along the coast. This analysis revealed a coherent low-frequency signal within the CUI time-series at 39°N (Figs. 3 and 6) that accounted for almost 10% of the total energy of the series.

This is the first report, to the best of our knowledge, of the presence of such a low-frequency signal in the CUI time series. Typically, the high-energy climatological annual cycle (i.e., the mean value for each time of the year) of the signal is removed from the series in the process of computing anomalies [31], [46]. These anomaly time-series have been compared with climatic indices but no significant correlations have been usually found [29], [30], [31], [46]. However, the low-frequency signal of the CUI time-series at 39°N was significantly correlated with each of the three major climatic indices we tested (Fig. 6).

Nevertheless, it is well known that upwelling processes in the CCS region vary regionally [44], so spatially integrated indices (e.g., globally or ocean-averaged SST time series) or a single index from an isolated location will not give a representative measure of its strength along the coast [20]. It makes sense, then, to extend this analysis to the CUI data for individual locations along the coast (Fig. 1). The energy distribution (Fig. 7) of the three signals consistently found within the different CUI time series (Table 1: annual, semiannual and low-frequency) allows us to divide the coast into three regions: south of 30°N, between 33 and 45°N, and north of 45°N.

The relatively low energy (40%) associated with the annual cycle south of 30°N agrees with previous observations, as no annual signal of upwelling-favorable winds south of 30°N was found using space-state models [20]. Also, it has been reported [47] that the most of the nutrient supply to the photic layer in the Southern California Bight occurs by geostrophically driven upwelling and winter mixing and not from seasonal coastal upwelling. Coastal chlorophyll observations in this region also show only a weak seasonal cycle and highly variable blooms [48].

This low energy of the annual signal and the relatively energetic (over 20%) low-frequency signal in the southern region indicates that the processes controlling the dynamics of this signal are much more important in the southern area than along the rest of the west coast. This agrees with previous results [49] suggesting that the southernmost region of the CCS is more affected by climatic low-frequency processes such as those related to ENSO and NPGO variability [50].

In the region immediately north (33°–39°N), coastal upwelling is known to be the main process governing seasonal fluctuations [41], [49]. Correspondingly, the energy of the annual signal for this region increased to an average of >75% while the energy associated with the semiannual and the low-frequency signals dropped to about 7% of total variability.

The strong decrease of the energy of the identified pure signals north of 45°N (Fig. 7) is accompanied by an increase of the variance associated with the non-structured variability (i.e., the noise of the series). This is likely related to the characteristic high turbulent mixing in this region [41] associated with the frequent passage of cyclones and storm systems [51], as has been previously observed for SST in this region [49].

It is also relevant to explore how the different signals of the CUI time series are correlated among the different locations. We computed the correlation coefficients between the different signals (total monthly CUI, annual signal and low-frequency signal) at every pair of locations. This analysis (data not shown) revealed that the total CUI and the annual cycle are very well correlated at all locations north of 27°N, indicating that the amplitudes and phases of both signals present coherent patterns throughout the CCS. However, the low-frequency signal is not coherent along the coast, indicating that the processes governing the dynamics of this signal could reverse phase or be altered from north to south (see relation with the climatic indexes below) or that local characteristics of the different sites make a strong difference in the signature of the dynamical response of this signal.

We compared the isolated low-frequency signals of the CUI with the different climatic indices known to influence the dynamics of this region to determine the areas that are more influenced by each climatic index (as in [52]). We also looked for the month with the maximum correlation, as this could provide clues of the underlying mechanisms responsible for the correlation patterns (Fig. 8a).

One common feature of the correlations between the low-frequency CUI signal and all the analyzed climatic indices is that the month with the strongest correlation always corresponded to spring-summer (March to September) south of 40°N, while north of this latitude higher correlations were found during late fall and winter (November to February) (Fig. 8a). It is known that November through March corresponds to the winter regime with low pressures in the Pacific, while the Pacific tends to be dominated more by the subtropical anticyclone for the rest of the year [53]. It appears then, that the CUI dynamics in the southern western coast are more influenced by subtropical variability while the northern part is more controlled by winter atmospheric patterns.

As expected, enhanced upwelling corresponds to low SST, hence the negative and significant correlation found for the majority of the locations along the western US coast (Fig. 8b). The same negative correlation pattern found for PDO (Fig. 8a) is consistent with its definition as the leading EOF of SST anomalies [26] and agrees well with the reported effect of PDO changes in the northeast Pacific [50]. Positive phases are related to a stronger Aleutian Low [26] and, thus, less intense equatorward winds along the western American coast [50]. Those winds should reduce coastal upwelling intensity, explaining the negative relationship found in our analysis for PDO and SST anomalies (Fig. 8a and 8b). This relationship has an immediate consequence for marine productivity [26]: positive PDO eras are associated with enhanced coastal ocean biological productivity in Alaska (above 60°N) and reduced productivity off the west coast southward [54]. Negative PDO eras show the opposite north-south pattern of marine ecosystem productivity [55].

**Figure 9:**
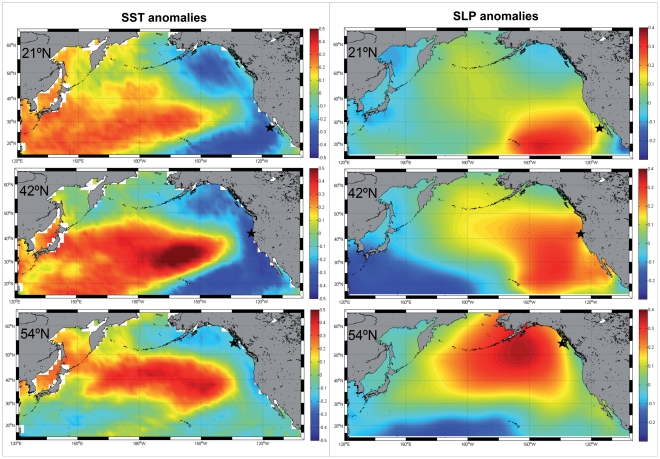
R-value distributions throughout the North Pacific. SST anomalies (left column) and SLP anomalies (right column) versus low frequency CUI signal at 27°N (upper panel), 42°N (central panel) and 54°N (lower panel). Black stars indicate the position of the CUI estimates.

To extend this analysis beyond the coastal zone we looked at the distribution of correlation coefficient between SST anomalies of the whole North Pacific basin and the CUI low-frequency signals at three representative locations of the coast (27°N, 42°N and 54°N) (Fig. 9, left panel). The distribution patterns were very similar to the PDO [26] but with opposite sign, the correlation being negative along the western North American coast and becoming positive in the central and eastern North Pacific. Correlation coefficients changed from −0.5 to +0.5 and were generally smaller when using the CUI from 54°N. These maps show that the negative correlation between PDO and the low-frequency CUI along the coast is consistent throughout the North Pacific, giving further support to the significance of the observed correlations along the coast.

The positive correlation with SLP at the coastal locations (Fig. 8b) was weaker and less significant that with SST, which seems to indicate that local atmospheric pressure conditions have limited impact on the upwelling characteristics. Therefore, to explore whether changes on basin-wide SLP anomalies (i.e., of the atmospheric patterns) were related to the low-frequency modulation of the CUI, we repeated the basin-wide spatial analysis of the correlation coefficients between SLP anomalies and CUI low-frequency at the same three locations of the coast (Fig. 9, right panel). In this case, maximum R-values were usually found at the same latitude where the CUI was calculated but a little farther off-shore, this probably being the reason for the low R-values found for the coastal locations (Fig. 8b). The highest R-values were found for the CUI at 54°N, which seems to be connected with the intensity of the Aleutian Low. As commented on above, it is known that a deeper Aleutian Low (i.e., negative anomalies of the SLP in this northern region) decreases equatorward winds along the western US coast [50], which leads to warm anomalies of SST as a consequence of the deactivation of the coastal upwelling [53], [56]. This is consistent with the observed distribution of R-values.

The inverse relationship with the ENSO index also agrees well with the reported effect of El Niño-La Niña cycles in the CCS [57]. Upwelling (and primary production) decreases during El Niño conditions, while enhanced upwelling and biological productivity have been reported during La Niña conditions [30]. Our results contrast, however, with those of [29], who found no correlation between the monthly anomalies of CUI and the MEI, and also with the later work of [32], who did not find a significant correlation between the second EOF mode of the CUI time series and the ELNINO2 index. These differences are likely due to the different protocols used to compute the low-frequency signal from the original time series in our study (SSA) compared to the previous studies (anomalies from the climatological annual cycle).

The fact that both PDO and MEI show similar correlation patterns is indicative of the atmospheric teleconnection pattern that drives changes in the Aleutian Low and consequently forces part of the PDO pattern [58]. In this sense, several authors [58] have proposed that the PDO can be considered to be the reddened response to both atmospheric noise and ENSO, resulting in more decadal variability than either. In fact, if SSA is applied to both climatic indices and the correlation coefficient between the two main signals within them (representing 61% of PDO and 72% of MEI) is computed, a value of R = 0.82 (p<0.01) is obtained, supporting this view. Therefore, it is not surprising that both indices show similar patterns of correlation with the CUI, as they are very strongly correlated themselves.

The positive correlation of the low-frequency CUI signal with the NPGO index south of 42°N agrees well with previous reports. It has been proposed that the NPGO reflects changes in wind stress in the CCS, in particular the winds that force coastal upwelling and open-ocean Ekman pumping [8]. Also, a positive phase of the index indicates an intensification of the North Pacific Current and subsequent reinforcement of the California Current, which (in conjunction with the intensified coastal winds) create upwelling-favorable conditions mainly in the region south of 38°N. This understanding fits quite well with the correlation pattern shown in Fig. 8a.

The shift in the correlation sign north of 42°N (Fig. 8a) reflects the bipolar nature of the climatic pattern represented by the NPGO index [8] and is entirely consistent with the associated wind pattern. Also [50] reported a change in the predominant direction of the wind field associated with the NPGO around 40°N. North of this latitude, positive NPGO is associated with poleward winds (reducing coastal upwelling). To the south, it is associated with more intense equatorward winds (i.e., increasing coastal upwelling).

The existence of the low-frequency signal in the CUI time series, and the fact that these signals are significantly correlated with different climatic indices, strongly suggest that the already known relationships between the Pacific (or global) climate fluctuations and the dynamics of the CCS are mainly driven by their effects on the magnitude and intensity of coastal upwelling. Our analyses have revealed the mechanisms for this connection. With this information, climatic indices can possibly be used more effectively to forecast or predict upwelling fluctuations in the CCS.
